# Brooke-Spiegler Syndrome: A Rare Entity

**DOI:** 10.1155/2014/231895

**Published:** 2014-01-23

**Authors:** Monika Rathi, Seema Awasthi, Satish Kumar Budania, Faiyaz Ahmad, Shyamoli Dutta, Ashutosh Kumar

**Affiliations:** ^1^Teerthanker Mahaveer Medical College and Research Center, Moradabad 244001, India; ^2^Lady Hardinge Medical College, New Delhi 110001, India

## Abstract

Brooke-Spiegler syndrome is a rare entity. It is an autosomal dominant syndrome in which multiple trichoepitheliomas, cylindromas, or other adnexal tumors are seen. Very few cases of Brooke-Spiegler syndrome are reported in the literature. We came across a 40 -year-old female in which multiple trichoepitheliomas and cylindromas were seen on scalp. In view of clinical history and histopathological examination it was diagnosed as Brooke-Spiegler syndrome. We report this case because of its rarity.

## 1. Introduction

Brooke-Spiegler syndrome was reported for the first time in 1842 by Ancell [1]. It is an autosomal dominantly inherited syndrome, characterised by multiple skin appendageal tumors [2]. More than 50 cases of Brooke-Spiegler syndrome are reported in the literature so far.

## 2. Case Report

**Figure 1:**
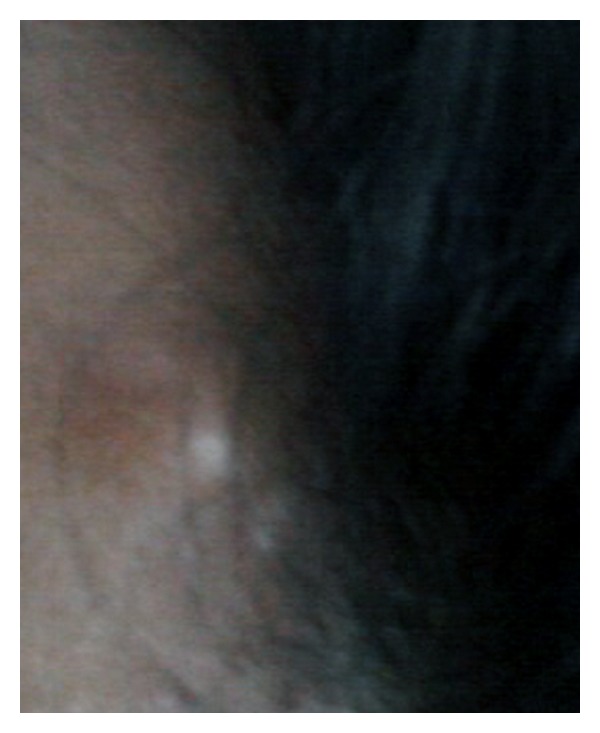
Papulonodular lesion on the scalp.

We report a case of 40-year-old female who presented to our hospital with the clinical complaint of multiple papulonodular lesions on the scalp, ranging in size from 0.2 × 0.2 cm to 2 × 2 cm for 1 year. Her mother also had similar lesions on face. She had no clinical symptoms associated with it. She came for cosmetic concerns and the gradual increase in size of the lesion. The largest lesion was excised and was sent to the histopathology department (Figure 1).

**Figure 2:**
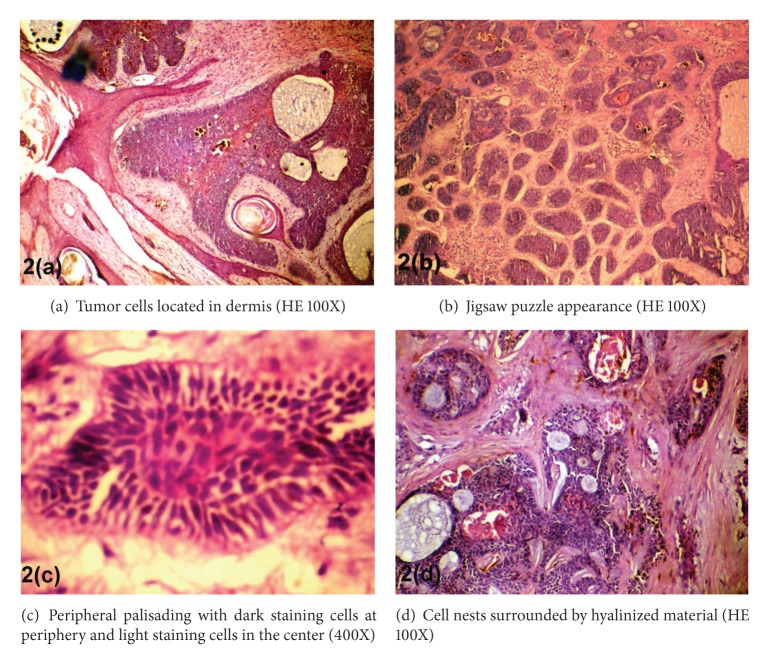
Histology of lesion.

**Figure 3:**
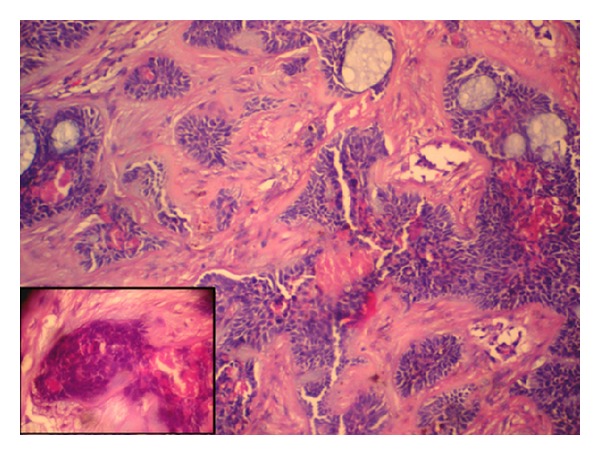
Cell nests, horn plugs, immature germ cells, and follicular papillae, surrounded by fibrotic and hyalinized stroma (HE 100X). Inset shows high power view of follicular papillae (HE 400X).

Gross-examination showed skin covered tissue. External surface showed the presence of some hairs. On microscopy, histopathological features of both trichoepithelioma and cylindroma were seen in the same lesion. Features favouring cylindroma included islands of basaloid cells surrounded by hyalinized material. These basaloid cells were arranged in jigsaw-puzzle-like architecture and enclosed lumina at few places. The cells in the periphery of islands were darker, and those in the center were lighter in color (Figure 2(a) to 2(d)). The histological feature suggestive of trichoepithelioma included epithelial elements arranged to form immature hair germ cells, papillary mesenchymal bodies known as follicular papillae, and horn cysts along with lace-like reticular basaloid structures (Figure 3). Thus, the diagnosis of collision tumor (cylindroma and trichoepithelioma) was made and a possibility of Brooke-Spiegler syndrome was suggested on the basis of history and histopathological examination.

## 3. Discussion

The association of multiple cylindromas, as an autosomal dominant disease with trichoepitheliomas, has been named Brooke-Spiegler syndrome [2].

In some cases of Brooke-Spiegler syndrome, multiple cylindromas, trichoepitheliomas, and spiradenomas are seen [3]. Brooke-Spiegler syndrome (BSS), familial cylindromatosis (FC), and multiple familial trichoepithelioma (MFT) share overlapping clinical findings. Patients with BSS are predisposed to multiple skin appendage tumors such as cylindroma, trichoepithelioma, and spiradenoma. FC, however, is characterized by cylindromas and MFT by trichoepitheliomas as the only tumor type [4].

Genetic studies have identified a single gene, CYLD1, on 16q12-q13 as being altered in Brooke-Spiegler syndrome [3, 5, 6]. The penetrance of the gene has been estimated to be between 60% and 100% [7]. However, mutations in CYLD1 gene are also found in familial cylindromatosis and familial trichoepithelioma. So, histopathology plays an important role to distinguish between BSS, FC, and MFT [4].

Our case presented with cylindroma and trichoepithelioma in the same lesion. Both the tumours are discussed in detail below.

Cylindroma is a benign basaloid tumor with folliculosebaceous distribution and controversial histogenesis. These are benign neoplasms with apocrine and eccrine differentiation [3]. Clinically, they may be solitary or multiple and usually occur in adults and increase in size throughout the life. They range in size from a few millimeters to several centimeters [3]. Solitary cylindromas are most common; these are erythematous or skin coloured lesions of the scalp, head, and neck or trunk. Some are painful. They may have overlying telangiectasia but otherwise are fleshy. Histologically, the tumors are circumscribed, nonencapsulated dermal nodules composed of islands and cords of basaloid cells surrounded by a thick, hyalinized, PAS-positive basement membrane. The cells are arranged in an interlocking “jigsaw-puzzle-” like architecture. Islands may have lumina or pseudolumina. Two cell types are described. The first is a small, dark cell, often located in the periphery of tumor nodule and the second is larger, lighter cell comprising the central portions of the cords [8].

Trichoepithelioma can exist in a familial or solitary form. The multiple form becomes apparent in adolescence or adulthood with predilection for central facial distribution [3].

The solitary form is most common and is seen in the head and neck region but can be found on any portion of hair bearing skin. They present as firm, elevated, flesh coloured nodules usually less than 2 cm in diameter. They are benign neoplasms with follicular differentiation [3]. Histologically, classic trichoepithelioma is a symmetric lesion that contains a mixture of epithelial elements ranging from hair germs associated with papillary mesenchymal bodies (follicular papillae) to small horn cysts and to lace-like reticular basaloid structures. The stroma containing these structures is typically fibrotic [8].

The modalities of treatment available for the adnexal tumors in BSS patients include excision of the tumor, dermabrasion, electrodessication, cryotherapy, and radiotherapy using argon and Co2 lasers. It has been shown that treatment with erbium-YAG laser causes less scars and fewer recurrences. It has been proven that the administration of aspirin and its derivatives can result in the rapid formation of new lesions [9]. In our case, lump was excised and since the patient was asymptomatic, she refused to go for any other modality of treatment. She was followed up to 1 year; she did not have any complaints.
